# Early discontinuation of chemotherapy in older adults with early breast cancer: results from the prospective multicenter HOPE study

**DOI:** 10.1093/jnci/djag250

**Published:** 2026-07-30

**Authors:** Jingran Ji, Can-Lan Sun, William Dale, Harvey Jay Cohen, Heeyoung Kim, Vani Katheria, Nikita Baclig, Yuliya Zektser, Arman Niknafs, Samuel Margolis, Catherine Lee, Clarie Smith, Mina S. Sedrak

**Affiliations:** 1 Department of Medicine, UCLA David Geffen School of Medicine, Los Angeles, CA, United States; 2 Center for Cancer and Aging, City of Hope, Duarte, CA, United States; 3 Center for the Study of Aging and Human Development, Duke University, Durham, NC, United States

## Abstract

**Purpose:**

Older adults with early-stage breast cancer (EBC) receiving neo/adjuvant chemotherapy frequently discontinue treatment early. However, the incidence, risk factors, and survival implications of early discontinuation remain poorly understood.

**Methods:**

This is a post hoc analysis of 501 older adults (aged ≥65) with EBC treated with neo/adjuvant chemotherapy from the Hurria Older PatiEnts (HOPE) Study. Before chemotherapy, we collected sociodemographic, clinical, and geriatric assessment characteristics. The primary outcome was early discontinuation (yes/no), defined as not completing all planned chemotherapy. We used multivariable logistic regression with stepwise selection to identify pretreatment risk factors and the Kaplan-Meier method to evaluate the impact of discontinuation on survival.

**Results:**

Of 501 participants (median [range] age, 70 [65-86] years), 113 (23%) had early discontinuation. After multivariable analysis, ≥1 fall within 6 months (odds ratio [OR]=2.06, 95% CI = 1.11 to 3.81, *P*=.02) and an activity of daily living score <85 (OR = 2.00, 95% CI = 1.26 to 3.18, *P*=.003) were identified as risk factors for early discontinuation. Participants with both risk factors were at highest risk (OR = 4.61, 95% CI = 2.18 to 9.79, *P*=.001). With a median follow-up of 4.3 years, early discontinuation was not associated with worse overall or breast cancer-specific survival but was associated with higher non-breast cancer-specific mortality (hazard ratio = 2.94, 95% CI = 1.22 to 7.07, *P*=.02).

**Conclusion:**

In this cohort of older adults with EBC, nearly one-quarter discontinued chemotherapy early. Early discontinuation was associated with increased non-cancer-related mortality but not cancer-related mortality, emphasizing the importance of identifying high-risk older adults and optimizing treatment delivery.

## Introduction

Adjuvant chemotherapy for women with high-risk early-stage breast cancer reduces the risk of recurrence and death from breast cancer, and this is true in both younger and older adults.^1–3^ Furthermore, studies have demonstrated that those who do not complete the full, planned chemotherapy regimen are more likely to have disease recurrence.^4–6^ This early discontinuation of chemotherapy is a problem frequently encountered among older adults, who commonly experience severe toxicities and are often unable to complete the prescribed treatment regimens.^7–9^

However, aging is heterogeneous, and not all older adults have early discontinuation of treatment. Two older adults with similar performance status may have different risks of not completing a prescribed treatment regimen. Currently, most studies examining treatment completion have focused on relative dose intensity (RDI), a metric that measures the percentage of the chemotherapy dose received relative to the total planned regimen dose.^5,6,10–13^ Patients who receive <85% of RDI have been shown to have an increased risk of disease recurrence and increased mortality.^11,14^ Among older adults with early breast cancer, we previously showed that older adults who receive <85% RDI have worse overall survival.^4^

However, there are limitations with the translation of RDI to clinical practice, given that the calculation can be difficult to carry out in real time, and there are no clear guidelines on how clinicians can use RDI to optimally counsel patients on treatment decisions. A more clinically relevant outcome to study may be early chemotherapy discontinuation, whether treatment was stopped before the planned number of cycles were delivered. Early discontinuation of adjuvant hormone therapy has been well described among patients with hormone receptor positive breast cancer, but very few studies have studied early discontinuation of chemotherapy.^15–18^ An analysis of the BQUAL study previously reported an early discontinuation rate of 12% among a young patient population, with the strongest risk factor as a regimen with >4 cycles of therapy.^19^ Moreover, no study to date has evaluated early treatment discontinuation among a cohort of older adults with early-stage breast cancer treated with neo/adjuvant chemotherapy.

Here, we examined the incidence, reasons, and risk factors for early discontinuation of neo/adjuvant chemotherapy in older adults with early breast cancer. We also evaluate the association between early discontinuation and survival outcomes in this population.

## Methods

The Hurria Older PatiEnts (HOPE) with Breast Cancer Study (NCT01472094) is a prospective multicenter study of older adults aged ≥65 with early-stage (I-III) breast cancer scheduled to receive standard-of-care neoadjuvant or adjuvant chemotherapy.^4,8,20–23^ The study was conducted by the Cancer and Aging Research Group (CARG) across 16 institutions in United States. All sites obtained institutional review board approval and written informed consent before enrolling study participants. Participants were assessed at baseline within 14days of treatment and followed throughout the treatment course. The study captured detailed geriatric, clinical, and treatment data for all study participants at baseline and rigorously documented treatment-related toxicity and chemotherapy dose modifications, including treatment discontinuation, throughout the treatment period. The primary analysis of the HOPE study was to develop and validate a risk prediction model for chemotherapy toxicity in older adults with early breast cancer.^8^ Here, we present a post hoc analysis focused on understanding the incidence, reasons for, and risk factors of early chemotherapy discontinuation and its association with survival outcomes.

### Study population

A total of 501 participants with stage I-III breast cancer were recruited and underwent neo/adjuvant chemotherapy from September 2011 to May 2017. All participants were included in our final analysis.

### Baseline measures

Before initiation of neo/adjuvant chemotherapy, we collected sociodemographic and clinical variables, including age, race/ethnicity, education, and marital status, cancer stage, hormone receptor status, history of prior radiation or chemotherapy, body mass index, body surface area, white blood cell count, hemoglobin, albumin, creatinine clearance, liver function [normal, defined as all liver function tests within normal range per institutional laboratory standards], and planned used of granulocyte-colony stimulating factor (G-CSF) prophylaxis (yes/no).

We also gathered baseline objective and self-reported measures of functional aging using the CARG geriatric assessment (GA).^4,8,20,23–25^ The CARG GA comprised of both health-care provider assessments and participant self-reported measures. The health-care provider portion includes measures of performance status (Karnofsky Performance Status^26^), physical function (Timed Up and Go Test^27^), cognitive screening (Blessed Orientation Memory Concentration^28^), and body composition measurements (height, weight, body mass index, unintentional weight loss). The participant portion included self-reported functional status (fall history, activities of daily living [ADL],^29,30^ instrumental ADL^31^), comorbidities,^32,33^ medications, psychological state (Mental Health Inventory-17),^34^ and social support.^35^

### Outcomes

Participants were followed from baseline, throughout chemotherapy, to 30 days after completion of treatment. The chemotherapy regimen was determined according to the discretion of the treating oncologist. Before starting chemotherapy, the planned regimen, number of cycles, and starting dose of treatment were documented. During treatment, the actual regimen received was recorded, including the number of drugs given each cycle and the number of cycles received for each drug. Chemotherapy cycles were defined in accordance with the standard regimen. For paclitaxel, the number of cycles were defined either as weekly for 12 cycles or every 2weeks for 4 cycles depending on the regimen they were associated with. These 2 regimens were analyzed together because the focus was on any discontinuation related to the drug irrespective of the dosing schedule. We also collected the incidence of grade 2 or higher chemotherapy-related toxicities, as graded by the National Cancer Institute Common Terminology Criteria for Adverse Events, version 4.0.

The primary endpoint was early discontinuation of chemotherapy, defined as a dichotomous variable (yes/no). Adapted from prior work in the literature,^36,37^ a patient was considered to have discontinued treatment early if 1 or more planned chemotherapy drugs were stopped at any time during the planned treatment course. Secondary endpoints for this post hoc analysis were overall survival, breast cancer–specific mortality, and non-breast-cancer-specific mortality. Overall survival time was calculated from the start of chemotherapy to the date of death due to any cause; the date of last contact; or December 31, 2019, whichever occurred first. Breast cancer–specific mortality was defined as death attributed to breast cancer, and non-breast-cancer mortality was defined as death from causes other than breast cancer. For both outcomes, time was measured from chemotherapy initiation to the date of the cause-specific death. Deaths from the alternate causes were treated as competing events, and patients without an event were censored at the date of last contact or December 31, 2019.

### Statistical analysis

To evaluate the risk factors associated with early discontinuation of chemotherapy, we first conducted univariate logistic regression analyses to examine the associations between pretreatment demographic, clinical, and GA variables and early discontinuation, as presented in Table 1. Variables with a *P* value <.05 in univariate analyses were entered into a stepwise multivariable logistic regression model to identify a parsimonious subset of variables independently associated with early discontinuation. Treatment setting (neoadjuvant vs adjuvant) was examined but was not significantly associated with early discontinuation. We included it as a covariate given its clinical relevance, and in the final model, we adjusted for age, cancer stage, treatment setting, and chemotherapy regimen.

Survival probabilities were estimated using the Kaplan-Meier method, and survival curves were compared using the log-rank test to assess the association between early treatment discontinuation and overall survival (OS). Hazard ratios (HRs) with 95% confidence intervals for OS were estimated using Cox proportional hazards models, adjusted for age, cancer stage, treatment setting, and chemotherapy regimen. For breast cancer-specific and non-breast cancer-specific death, competing risks analyses were conducted using the Fine and Gray subdistribution hazards model, and cause-specific hazard ratios adjusted for age, cancer stage, treatment setting, and chemotherapy regimen were reported. All analyses were conducted using SAS version 9.4 (SAS Institute Inc., Cary, NC).

## Results

### Patient characteristics

Of the 501 participants, the median (range) age was 70 (65-86), and most (57%) were between the ages of 65 and 70 (Table 1). Most participants were female (99%), were non-Hispanic White (74%), had a college degree or higher (71%), and were married (55%). In terms of disease and treatment characteristics, most had stage II or III disease (65%), 47% had hormone-positive, human epidermal receptor 2 (HER2) negative, and most received chemotherapy in the adjuvant setting (81%).

Chemotherapy regimens were divided into 6 categories. The most frequently used regimens were anthracycline-based + no trastuzumab (32%) and non-anthracycline-based + no trastuzumab (36%). Less common regimens included anthracycline-based + trastuzumab (5%), platinum or CMF (cyclophosphamide, methotrexate, fluorouracil) + trastuzumab (10%), paclitaxel only + trastuzumab (11%), or other (6%, did not fit into any of the preceding categories). A total of 97.5% of participants received the guideline-concordant standard upfront treatment dose. Most participants received primary G-CSF prophylaxis (72%). Participants who received neoadjuvant chemotherapy received more anthracycline-based regimens and had a longer duration of treatment (Table 2).

The mean (SD) Karnofsky Performance Status was 93 (9.3). The median (range) number of comorbidities was 2 (0-11). In the geriatric assessment, 51% noted impairments in ADL, and 24% had impairments in instrumental ADL. Sixty (12%) participants had a fall in the 6 months before chemotherapy, and 212 (43%) noted that they could not walk more than 1 mile. The 3 most common comorbidities were hypertension (58.1%), arthritis (50.7%), and diabetes (20.2%). In total, 72% of patients reported living with someone (eg, such as partner or caregiver). A minority of participants (28%) endorsed limited social activity, although the majority (67%) endorsed limited social support.

### Incidence and reasons for early discontinuation

**Figure 1.**
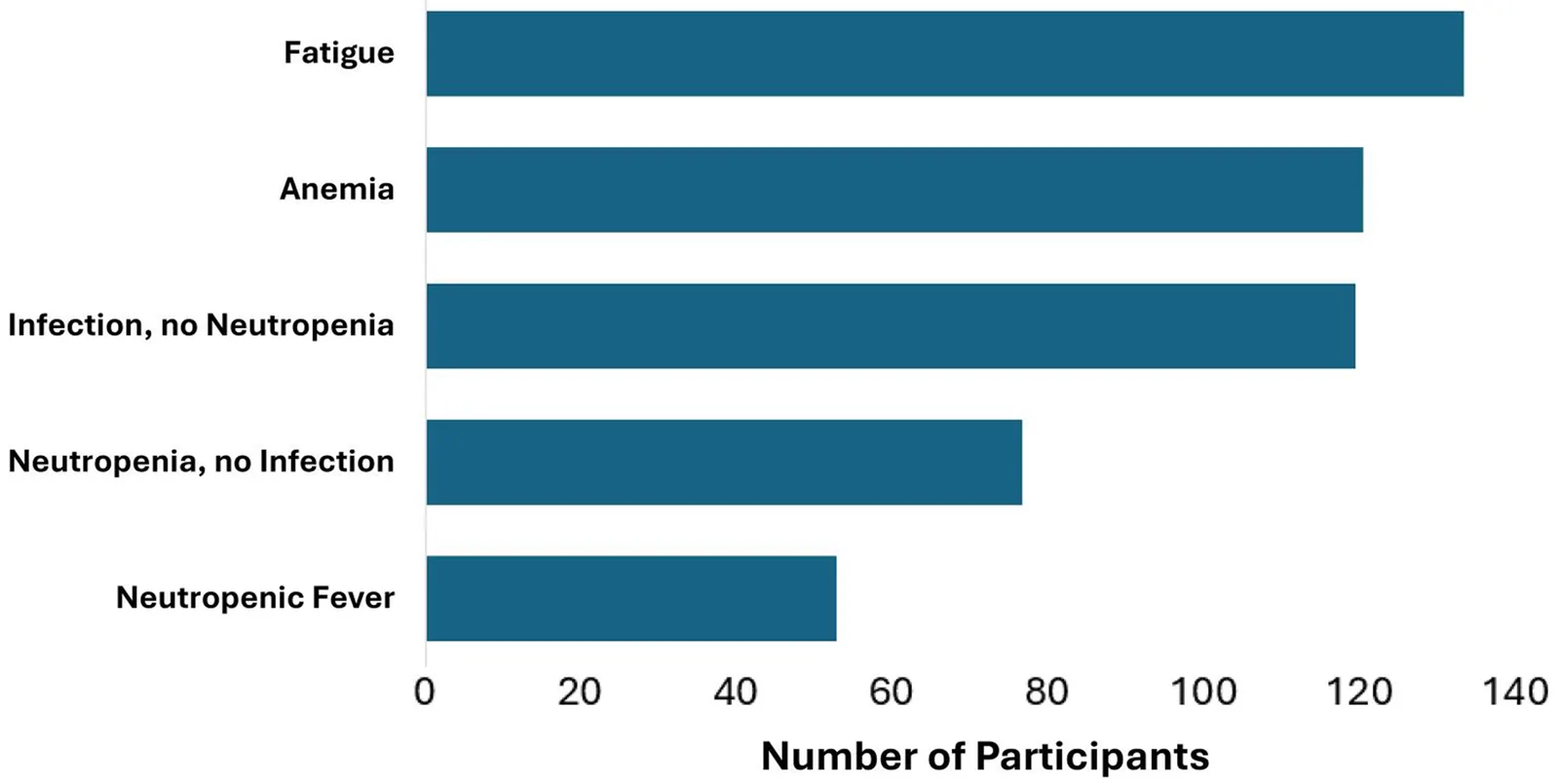
Most common grade 3+ chemotoxicities among those with early discontinuation.

**Figure 2.**
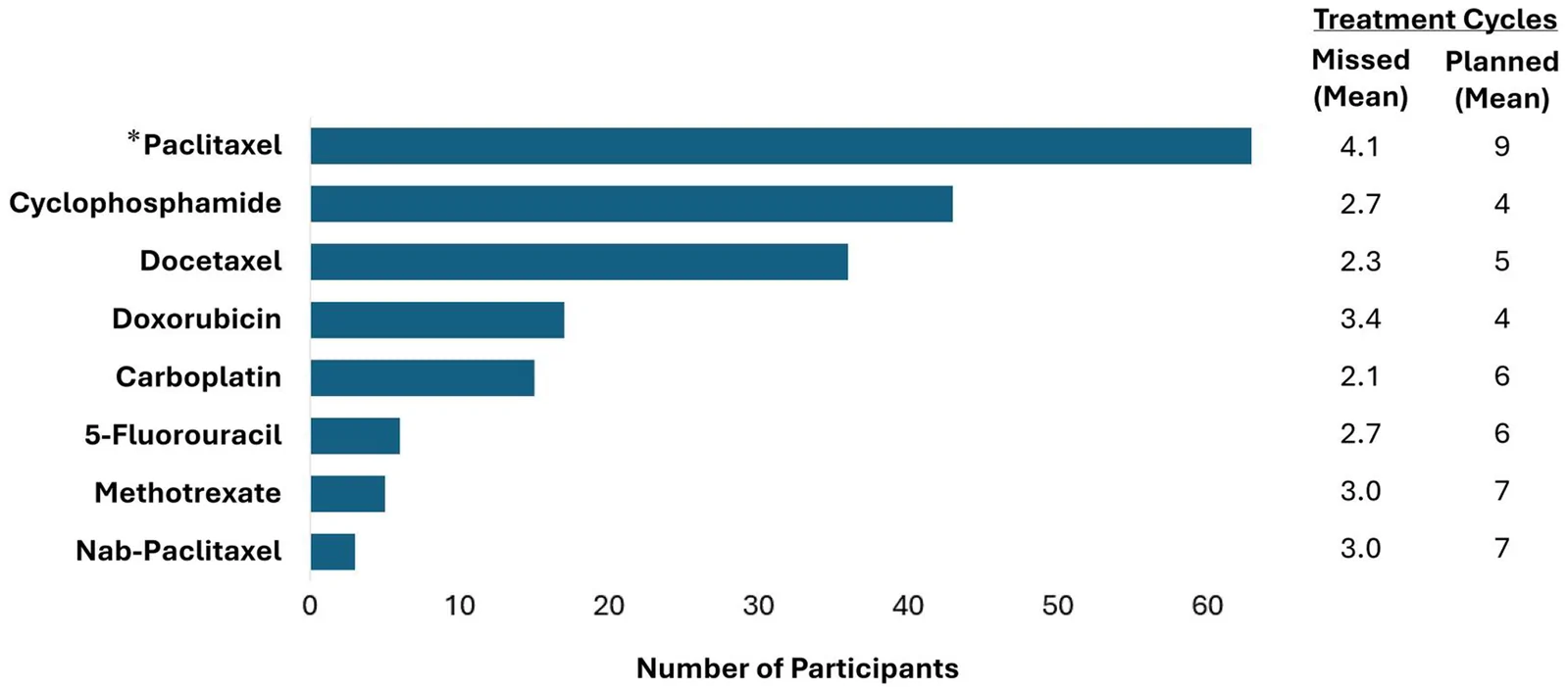
Most commonly discontinued chemotherapy agents and mean number of cycles missed. *Paclitaxel was given either weekly for 12 cycles or every 2 weeks for 4 cycles depending on the regimen that it was associated with.

A total of 113 (23%) participants had early discontinuation. All participants (100%) who had early discontinuation experienced at least 1 grade 2+ toxicity (Table 3), and the majority (85%) had at least 1 grade 3 or higher toxicity. Participants who received neoadjuvant therapy had more grade 3 and 4 toxicities compared with those who received adjuvant therapy (Table 2). There were 4 grade 5 toxicities recorded during the study period. Two participants who did not discontinue therapy early died from infection and thromboembolism, respectively. One participant who discontinued therapy early died from colon perforation, and another died from an unspecified cause. Among the participants with early discontinuation, the most commonly reported grade 3+ toxicities were fatigue, anemia, infection without neutropenia, neutropenia without infection, and neutropenic fever (Figure 1). The most frequently discontinued chemotherapy drug was paclitaxel, followed by cyclophosphamide and docetaxel (Figure 2). The majority of drugs (73%) were stopped within 3 cycles to the end of treatment.

### Risk factors associated with early discontinuation of chemotherapy

In univariate analysis, early discontinuation was associated with older age (continuous), higher stage (II and III), use of chemotherapy regimens that did not fall into the non-anthracycline+no trastuzumab category, lower albumin (continuous), kidney dysfunction, having a diagnosis of diabetes, patient-reported falls in the last 6months, ADL score of <85, and an instrumental ADL score of <14 (Table 4). No other baseline characteristics as listed in Table 1 showed statistical significance on univariate analysis. After multivariable stepwise logistic regression selection, only chemotherapy regimen, ADL score <85, and falls in the last 6months retained statistical significance.

In the final multivariable model, after adjusting for age, stage, treatment setting, and chemotherapy regimen, participants with an ADL score <85 (odds ratio [OR]=2.00, 95% CI = 1.26 to 3.18, *P*=.003) and those with a history of falls in the last 6 months (OR = 2.06, 95% CI = 1.11 to 3.81, *P*=.02) had significantly higher odds of early discontinuation (Table 5). When evaluated jointly, compared with participants with no falls and ADL ≥85, those with no falls but ADL <85 had significantly higher odds of early discontinuation (OR = 1.80, 95% CI = 1.10 to 2.96; *P*=.02). The highest odds were observed among participants with both a history of falls and ADL <85 (OR = 4.61, 95% CI = 2.18 to 9.79; *P*<.001). In contrast, those with a fall history but preserved ADL (≥85) did not have significantly increased odds (OR = 1.08, 95% CI = 0.29 to 4.07; *P*=.91).

### Early discontinuation and survival

**Figure 3.**
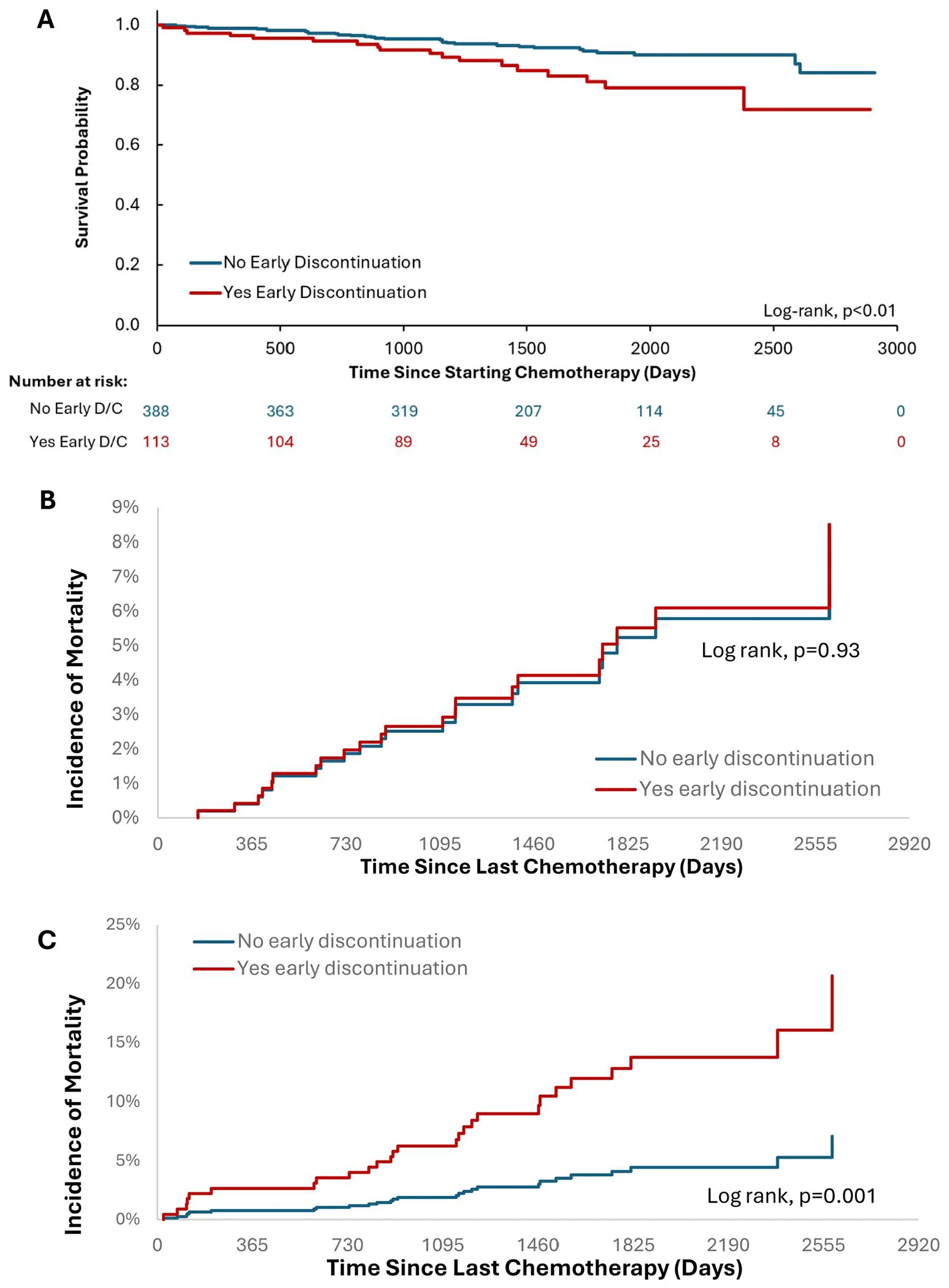
Unadjusted (**A**) Overall Survival, (**B**) Cumulative Incidence of Breast Cancer-Specific Mortality, and (**C**) Cumulative Incidence of Non-Breast Cancer-Specific Mortality by Treatment Discontinuation status.

After a median follow-up of 4.3 years (range = 24 days to 8.0years), 49 patients had died, 22 due to breast cancer and 27 from non-breast-cancer-related causes (Table S1). Of the participants who had breast cancer recurrence, there was no difference between rates of local and distant recurrence (Table S2). Univariately, compared with patients who completed treatment, those with early discontinuation had significantly worse overall survival (log-rank *P*<.01; Figure 3A). Univariate cumulative incidence curves showed no significant difference in breast cancer-specific death between groups (*P*=.93), whereas the cumulative incidence of non-breast cancer death was significantly higher in the early discontinuation group (*P*=.001; Figure 3B and C). In adjusted analyses accounting for age, cancer stage, treatment setting, and chemotherapy regimen, early discontinuation was significantly associated only with increased non-breast-cancer mortality (HR = 2.94, 95% CI = 1.22 to 7.07, *P*=.02). After adjustment, early discontinuation was not significantly associated with breast cancer–specific mortality (HR=.91, 95% CI = 0.29 to 2.89, *P*=.87) or overall mortality (HR = 1.77, 95% CI = 0.96 to 3.25, *P*=.07).

## Discussion

In this post hoc analysis of a multicenter prospective cohort, nearly 1 in 4 older adults with early breast cancer treated with standard neo/adjuvant chemotherapy were unable to complete the planned treatment regimen yet did not have increased breast cancer-specific mortality compared with those who completed treatment. All participants who discontinued therapy early had at least 1 grade 2+ toxicity, and most had a grade 3+ toxicity. This study further identified that the most significant risk factors for early discontinuation were a baseline ADL score <85 and a recent history of falls before starting treatment.

Our study has several implications. First, our findings suggest that stopping chemotherapy early in older adults for treatment- related toxicity may not adversely affect breast cancer-specific survival. In fact, having to discontinue treatment may instead be an indicator of excess toxicity and poor tolerance to chemotherapy, leading to a higher risk of overall mortality due to competing causes of death other than breast cancer even after treatment has been stopped. For example, we know that functional decline after breast cancer treatment leads to increased mortality in older survivors of breast cancer.^38^ This emphasizes the importance of identifying these high-risk patients earlier, ensuring careful treatment selection and implementation of preemptive dose reductions for even mild toxicities to mitigate the non-breast cancer mortality risk in those who are unable to finish the full intended course of treatment.

Second, clinicians should include an assessment of functional variables such as those commonly collected in a geriatric assessment before initiating chemotherapy treatment in older adults. The risk factors for discontinuation that we identified, falls and low ADL score, are both markers of physical function impairment, suggesting that these patients are likely to have worse functional reserve even before exposure to chemotherapy. We have previously found that a positive fall history before chemotherapy is associated with a higher risk of chemotherapy toxicity-related hospitalizations.^20^ Hospitalizations due to toxicity are likely closely correlated with subsequent treatment discontinuation, given the severity of the related toxicity. Having a positive fall history is also likely indicative of global dysfunction at baseline.^39^ Similarly, having a low ADL score is a measure of physical function impairment. ADL is a self-reported measure that captures basic self-care independence (eg, walking, bathing, dressing) on a scale of 0-100. Individuals with higher ADL scores (a score of >85) have increased morbidity and improved all-cause survival compared with those with lower ADL scores.^40–43^ Moreover, lower ADL scores have similarly been associated with more chemotherapy toxicity, decreased tolerability to treatment, and lower rates of treatment completion.^44,45^ Thus, participants with both a positive fall history and a low ADL score may have impairments in multiple overlapping systems and are thus more susceptible to treatment toxicity. Identifying these vulnerable patients early may also allow for targeted interventions such as tailored exercise programs or physical therapy regimens before and during treatment to improve function, minimize treatment discontinuation, and improve survival.^46–49^

Third, our findings indicate that nearly one-fourth of older patients fail to complete their planned treatment. Our results are consistent with another cohort that reported an early discontinuation rate as high as 28%^37^ and an additional study that reported a rate of 21%.^36^ However, our findings differ from the BQUAL study, which reported a rate of about 11%.^19^ There are likely two reasons for this difference: (1) The patient population is much older in our cohort (median age = 70 vs 54.5) compared with the BQUAL cohort and (2) our definition of early discontinuation included participants who had early discontinuation of 1 chemotherapy drug at any time, whereas the BQUAL study used a definition of receipt of <80% of the number of planned chemotherapy cycles. However, all studies to date, including ours, were secondary analyses of prospective data or retrospective studies with a limited follow-up duration (<10years). Future research is needed to prospectively evaluate the incidence and impact of early discontinuation in older adults with early breast cancer, especially vulnerable patients with competing risk factors.

There were several limitations to our study. First, our definition of early treatment discontinuation was broad and included all participants who did not complete the planned number of cycles for any drug without any specification on the number of cycles missed. This approach allowed for some heterogeneity in the outcome measure as some patients only missed 1 cycle, whereas others missed more than 4 cycles of treatment, which likely has a different impact on outcomes. Second, the participants in this study were all deemed to be fit for chemotherapy by their treating oncologists and most had a physician-rated KPS ≥90, which may not be representative of the general older adult population. Third, our median follow-up time was 4.3years, which is relatively short given the substantially longer median overall survival in this patient population. Fourth, although we did record the most common toxicities associated with early discontinuation, we cannot definitively identify the specific reason that chemotherapy was discontinued, because many times it was multifactorial and likely not attributed to 1 toxicity alone. Fifth, data collection stopped at the end of chemotherapy, so no data on subsequent adjuvant therapies such as endocrine therapy or HER2-targeted therapy were collected, which could have further informed the reasons for disease recurrence and survival. Sixth, the multiagent chemotherapy plus trastuzumab category included a very small number of participants who received a nonstandard CMF + trastuzumab regimen. Although this introduces some heterogeneity into the group, sensitivity analysis confirmed that the exclusion of these participants did not alter the direction, magnitude, or statistical significance of any study outcomes. Seventh, most of the sites were academic centers located in an urban setting. Although many of these centers had community practice sites, this study population does not adequately capture toxicity management resources of rural, low economic, limited caregiver support, and low education community settings. Eighth, we did not collect data on payor status or economic status of the participants and thus are unable to evaluate the impact of financial distress on the likelihood of early termination. Furthermore, we did not incorporate measures of health literacy (eg, patient understanding of the implications of early discontinuation), assess dose intensity (eg, the impact of stopping treatment earlier vs later on in the treatment course), or evaluate the association between early discontinuation and the composite measures such as the CARG-BC toxicity score or the CARG GA frailty measure. Finally, this was a secondary analysis of the original study, and future prospective studies are necessary to validate our findings.

## Conclusion

Almost 1 in 4 older adults with early-stage breast cancer treated with neo/adjuvant chemotherapy had early discontinuation of treatment. Pretreatment assessment of falls and functional status may provide a simple and clinically actionable approach to identifying patients at increased risk for early chemotherapy discontinuation. Integrating these measures into routine care could enhance individualized treatment decision making and enable timely implementation of geriatric-informed supportive interventions. Future studies should evaluate whether addressing these vulnerabilities can improve treatment completion, preserve dose intensity, and ultimately enhance cancer outcomes in this growing and vulnerable patient population.

## Supplementary Material

Supplementary Material

Supplementary material is available at *JNCI: Journal of the National Cancer Institute* online.

## Figures and Tables

**Table 1.** Baseline characteristics of older women with early breast cancer, overall and by early discontinuation status.

Characteristic	Early discontinuation		Overall *N* = 501^a^ No. (%)
	No *n* = 388^a^ No. (%)	Yes *n* = 113^a^ No. (%)	
Age, years, median (range)	69.5 (65-86)	70.0 (65-86)	70.0 (65-86)
Categorical age group			
65-70	227 (58.5)	59 (52.2)	286 (57.1)
≥71	161 (41.5)	54 (47.8)	215 (42.9)
Female	385 (99.2)	112 (99.1)	497 (99.2)
Race/Ethnicity			
White, non-Hispanic	292 (75.3)	80 (70.8)	372 (74.3)
Black	60 (15.5)	17 (15.0)	77 (15.4)
Others	36 (9.3)	16 (14.2)	52 (10.4)
Education			
High school diploma or less	104 (27.1)	38 (33.9)	142 (28.6)
College degree	166 (43.2)	48 (42.9)	214 (43.1)
Post-college	114 (29.7)	26 (23.2)	140 (28.2)
Marital status			
Married	219 (56.7)	56 (50.0)	275 (55.2)
Other	167 (43.3)	56 (50.0)	223 (44.8)
AJCC stage^b^			
I	147 (37.9)	30 (26.5)	177 (35.3)
II/III	241 (62.1)	83 (73.5)	324 (64.7)
Hormone receptor (HR) status			
HR negative, HER2 negative	100 (25.8)	27 (23.9)	127 (25.3)
HR positive, HER2 negative	189 (48.7)	47 (41.6)	236 (47.1)
HR negative, HER2 positive	32 (8.2)	13 (11.5)	45 (9.0)
HR positive, HER2 positive	67 (17.3)	26 (23.0)	93 (18.6)
Prior therapies, pre-enrollment			
History of prior radiation	20 (5.2)	2 (1.8)	22 (4.4)
History of prior chemotherapy	19 (4.9)	3 (2.7)	22 (4.4)
Chemotherapy treatment setting			
Neoadjuvant	71 (18.3)	28 (24.8)	99 (19.8)
Adjuvant	317 (81.7)	85 (75.2)	402 (80.2)
Chemotherapy treatment regimen			
Anthracycline based + No Trastuzumab	120 (30.9)	40 (35.4)	160 (31.9)
Non-Anthracycline based + No Trastuzumab	157 (40.5)	25 (22.1)	182 (36.3)
Anthracycline based + Trastuzumab	18 (4.6)	7 (6.2)	25 (5.0)
Platinum/CMF + Trastuzumab^d^	32 (8.2)	17 (15.0)	49 (9.8)
Taxol only + Trastuzumab	41 (10.6)	16 (14.2)	57 (11.4)
Other	20 (5.2)	8 (7.1)	28 (5.6)
Polychemotherapy			
Yes	336 (86.6)	92 (81.4)	428 (85.4)
No	52 (13.4)	21 (18.6)	73 (14.6)
Planned treatment duration			
≤12 weeks (3 months)	200 (51.5)	42 (37.2)	242 (48.3)
>12 weeks (3 months)	188 (48.5)	71 (62.8)	259 (51.7)
Up-front dose reduction			
Yes	8 (2.1)	5 (4.4)	13 (2.6)
No	380 (97.9)	108 (95.6)	488 (97.4)
Standard regimen			
Yes	368 (94.8)	102 (90.3)	470 (93.8)
No	20 (5.2)	11 (9.7)	31 (6.2)
Planned supportive intervention			
Primary G-CSF prophylaxis	280 (72.2)	78 (69.0)	358 (71.5)
Pretreatment organ function			
WBC (103 cells/mm3), mean (SD)	9.2 (9.3)	7.4 (2.7)	8.7 (8.4)
Hemoglobin (g/dL), mean (SD)	13.0 (1.2)	12.9 (1.4)	12.9 (1.3)
Albumin (g/dL), mean (SD)	4.4 (2.07)	4.1 (0.50)	4.3 (1.80)
Normal liver function	348 (90.4)	95 (84.8)	443 (89.1)
Kidney, GFR ≥60	321 (84.0)	85 (75.2)	406 (81.0)
Performance status			
Physician-rated KPS, mean (SD)	93.6 (9.2)	92.5 (9.8)	93.3 (9.3)
Comorbidities			
No. of comorbidities, median (range)	2 (0-11)	2 (0-8)	2 (0-11)
Hypertension	229 (59.0)	62 (54.9)	291 (58.1)
Arthritis	196 (50.5)	58 (51.3)	254 (50.7)
Diabetes	70 (18.0)	31 (27.4)	101 (20.2)
Osteoporosis	79 (20.4)	18 (15.9)	97 (19.4)
Depression	66 (17.0)	25 (22.1)	91 (18.2)
Cardiovascular disease	36 (9.3)	14 (12.4)	50 (10.0)
Nutrition			
Obesity (BMI ≥30)	163 (42.0)	56 (49.6)	219 (43.7)
Body surface area mean, m2 (SD)	1.8 (0.2)	1.9 (0.2)	1.8 (0.2)
Physical function			
No. of falls in last 6months, median (range)	1 (1-4)	1.5 (1-6)	1 (1-6)
≥1 Fall in the last 6months	38 (9.9)	22 (19.6)	60 (12.1)
Timed up and go (seconds), mean (SD)	11.5 (6.0)	12.2 (5.6)	11.6 (5.9)
Unable to walk >1mile	151 (39.6)	61 (54.0)	212 (42.9)
ADL score <85^c^	183 (47.4)	72 (63.7)	255 (51.1)
IADL score <14	82 (21.3)	35 (31.5)	117 (23.6)
Cognitive function			
BOMC score ≥11	5 (1.3)	2 (1.8)	7 (1.4)
Psychological status			
Depression score ≥85	235 (61.2)	62 (56.9)	297 (60.2)
Anxiety score ≥85	168 (43.8)	47 (43.1)	215 (43.6)
MHI-17 Total score, ≥85	180 (46.9)	46 (42.2)	226 (45.8)
Social factors			
Limited social activity <60	105 (27.3)	35 (31.8)	140 (28.3)
Limited social support <100^e^	259 (67.4)	73 (64.6)	332 (66.8)

Abbreviations: ADL = activities of daily living (Medical Outcomes Study [MOS] subscale); AE = adverse event; AJCC = American Joint Committee on Cancer; BMI = body mass index; BOMC = Blessed Orientation Memory Concentration Test; G-CSF = granulocyte colony stimulating factor; GFR = glomerular filtration rate (calculated using Cockcroft-Gault Formula, with ideal body weight); HER2 = human epidermal growth factor receptor 2; HR = hormone receptor; IADL = instrumental activities of daily living (Older American Resources and Services [OARS] subscale); KPS = Karnofsky performance status; MHI-17 = Mental Health Inventory-17; WBC = white blood count.

a Some patients had missing information: 5 missing education, 3 missing marital status, 4 missing liver function, 5 missing creatinine clearance, 1 missing KPS, 4 missing fall history, 7 missing walk capacity, 2 missing ADL, 5 missing IADL, 7 missing BOMC, 8 missing MHI, 6 missing social activity, and 4 missing social support.

b Stage reflects pathological stage for patients treated in the adjuvant setting and clinical stage for patients treated in the neoadjuvant setting.

c The ADL score was calculated using the Physical Functioning Scale (PF-10) from the Medical Outcomes Study. The PF-10 assesses performance using self-reported ability to performe 10 activities: vigorous activities, moderate activities, lifting/carrying groceries, climbing several flights of stairs, climbing 1 flight of stairs, bending/kneeling/stooping, walking more than a mile, walking several blocks, walking 1 block, and bathing/dressing. Each item is rated on a 3-point scale based on limitation to perform that item: (1) Yes, limited a lot; (2) Yes, limited a little; and (3) No, not limited at all. Raw scores are summed and then transformed to a 0-100 scale, where 0 represents the lowest level of functioning and 100 represents no limitations.

d A small number of participants received CMF plus trastuzumab. These participants were included with those who received a platinum drug plus trastuzumab (TCHP) because both are trastuzumab regimens with multiagent chemotherapies.

e Social support was evaluated using the Medical Outcomes Study Social Support Survey, which contains 19 items assessing the perceived availability of 4 functional dimensions of social support. Each item is rated on a 5-point Likert scale: 1 = none of the time, 2 = a little of the time, 3 = some of the time, 4 = most of the time, 5 = all of the time. Scores were transformed to a 0-100 scale, and <100 was considered limited social support.

**Table 2.** Comparison of key characteristics between participants with neoadjuvant vs adjuvant chemotherapy.

Characteristic	Neoadjuvant *n* = 99 No. (%)	Adjuvant *n* = 402 No. (%)	*P*
Early discontinuation	28 (28.3)	85 (21.1)	.13
Chemotherapy regimen			<.01
Anthracycline based + No Trastuzumab	46 (46.5)	114 (28.4)	
Non-Anthracycline based + No Trastuzumab	15 (15.2)	167 (41.5)	
Anthracycline based + Trastuzumab	9 (9.1)	16 (4.0)	
Platinum/CMF + Trastuzumab	9 (9.1)	40 (10.0)	
Taxol only + Trastuzumab	6 (6.1)	51 (12.7)	
Other	14 (14.1)	14 (3.5)	
Planned treatment duration			<.01
≤12 weeks (3 months)	25 (25.3)	217 (54.0)	
>12 weeks (3 months)	74 (74.7)	185 (46.0)	
Adverse events			<.01
No Grade 2+ adverse events	13 (13.1)	69 (17.2)	
Grade 2 adverse event	30 (30.3)	160 (39.8)	
Grade 3 adverse event	37 (37.4)	142 (35.3)	
Grade 4 adverse event	16 (16.2)	31 (7.7)	
Grade 5 adverse event	3 (3.0)	0 (0)	
Baseline functional characteristics			
>1 fall in the last 6months	15 (15.3)	45 (11.3)	.28
ADL score <85	50 (50.5)	194 (48.5)	.72

Abbreviations: ADL = activities of daily living (Medical Outcomes Study [MOS] subscale); CMF = cyclophosphamide, methotrexate, fluorouracil.

**Table 3.** Rates of grade 2+ chemotherapy-related toxicity.

	Early discontinuation		Overall *N* = 501 No. (%)
	No *n* = 388^a^ No. (%)	Yes *n* = 113^a^ No. (%)	
Chemotherapy toxicity			
No Grade 2+ Adverse Events	82 (21.1)	0 (0)	82 (16.4)
Grade 2 Adverse Event	173 (44.6)	17 (15.0)	190 (37.9)
Grade 3 Adverse Event	110 (28.4)	69 (61.1)	179 (35.7)
Grade 4 Adverse Event	21 (5.4)	25 (22.1)	46 (9.2)
Grade 5 Adverse Event	2 (0.5)	2 (1.8)	4 (0.8)

**Table 4.** Baseline variables significantly associated with early discontinuation in univariate logistic regression analysis.

Baseline Characteristic	OR (95% CI)	*P*
Age, years (continuous)	1.04 (0.997 to 1.09)	.07
AJCC stage		
I	1.00	
II/III	1.69 (1.06 to 2.69)	.03
Chemotherapy regimen		
Non-Anthracycline based + No Trastuzumab	1.00	
Anthracycline based + No Trastuzumab	2.09 (1.20 to 3.64)	.009
Anthracycline based + Trastuzumab	2.44 (0.93 to 6.44)	.07
Platinum/CMF + Trastuzumab	3.34 (1.62 to 6.88)	.001
Taxol only + Trastuzumab	2.45 (1.20 to 5.01)	.01
Other	2.51 (1.00 to 6.32)	.05
Albumin (continuous)	0.42 (0.23 to 0.75)	.004
Kidney function (COCKCROFT)		
> =60	1.00	
<60	1.73 (1.04 to 2.88)	.03
Comorbidity—diabetes		
No	1.00	
Yes	1.72 (1.06 to 2.80)	.03
Falls in the last 6 months		
No	1.00	
Yes	2.23 (1.25 to 3.95)	.006
IADL		
14	1.00	
<14	1.70 (1.07 to 2.72)	.03
ADL		
≥85	1.00	
<85	1.95 (1.26 to 3.00)	.003

Abbreviations: ADL = activities of daily living (Medical Outcomes Study [MOS] subscale); AJCC = American Joint Committee on Cancer; CMF = cyclophosphamide, methotrexate, 5-Fluorouracil; IADL = instrumental activities of daily living (Older American Resources and Services [OARS] subscale).

**Table 5.** Association between baseline characteristics and early discontinuation.

	Early Discontinuation			Multivariable^b^	
	Yes (*n* = 113)	No (*n* = 388)	Total^a^ (*N* = 501)	OR (95% CI)	*P*
**≥1 fall in last 6 months**					
No	90 (20.6%)	346 (79.4%)	436	1.00	
Yes	22 (36.7%)	38 (63.3%)	60	2.06 (1.11 to 3.81)	.02
**ADL score**					
≥85	41 (16.8%)	203 (83.2%)	244	1.00	
<85	72 (28.2%)	183 (71.8%)	255	2.00 (1.26 to 3.18)	.003
**Combination of falls and ADL score**					
No Falls/ADL Score ≥85	37 (16.6%)	186 (83.4%)	223	1.00	
Yes Falls/ADL Score ≥85	3 (16.7%)	15 (83.3%)	18	1.08 (0.29 to 4.08)	.91
No Falls/ADL Score <85	53 (25.0%)	159 (75.0%)	212	1.80 (1.10 to 2.96)	.02
Yes Falls/ADL Score <85	19 (45.2%)	23 (54.8%)	42	4.61 (2.18 to 9.79)	<.001

Abbreviations: ADL = activities of daily living (Medical Outcomes Study [MOS] subscale); CI = confidence interval; OR = odds ratio.

a Four patients missing fall information; one patient missing both ADL score and fall information.

b Adjusted for age (continuous), stage (I vs II/III), treatment setting (neoadjuvant vs adjuvant), and regimen (anthracycline based + no trastuzumab vs non-anthracycline based + no trastuzumab vs anthracycline based + trastuzumab vs platinum/CMF + trastuzumab vs taxol only + trastuzumab vs other).

## Data Availability

A deidentified dataset is available upon request via a Data Request Form that can be obtained from Vani Katheria (vkatheria@coh.org), Scientific Program Manager of the Center for Cancer and Aging at the City of Hope, Duarte, California. The purpose of the form is to register a request, not to collect information to be used in deciding whether the request has merit.
