# Supplementary material for: Early discontinuation of chemotherapy in older adults with early breast cancer: results from the prospective multicenter HOPE study
Source: J Natl Cancer Inst. Author manuscript; Available in PMC 2026 Sep 27. (PMC13616576; doi:10.1093/jnci/djag250)
Supplement: Supplementary file 1 [file NIHMS2210349-supplement-Supplementary_Material.docx]

**SUPPLEMENTARY MATERIALS**

**Early Discontinuation of Chemotherapy in Older Adults with Early Breast Cancer: Results from the Prospective Multicenter HOPE Study**

Jingran Ji, Can-Lan Sun, William Dale, Harvey Jay Cohen, Heeyoung Kim, Vani Katheria, Nikita Baclig, Yuliya Zektser, Arman Niknafs, Samuel Margolis, Catherine Lee, Clarie Smith, Mina S. Sedrak

**Contents**

**Table S1.** Causes of death in older patients (65+) with early stage breast cancer who did and did not discontinue neoadjuvant or adjuvant chemotherapy earlier than planned.

**Table S2.** Rates of locoregional and distant recurrence among patients who did and did not discontinue neoadjuvant or adjuvant chemotherapy earlier than planned.

**Table S1**. Causes of death in older patients (65+) with early stage breast cancer who did and did not discontinue neoadjuvant or adjuvant chemotherapy earlier than planned.

|  | **Early Discontinuation** | |
| --- | --- | --- |
|  | **No**  **n=388**  **No.** | **Yes**  **n=113**  **No.** |
| **Breast Cancer Related Death** | **17** | **5** |
| **Non-Breast Cancer Related Death** | **14** | **13** |
| Acute Kidney Failure | 1 | 0 |
| Complications post-treatment | 2 | 1 |
| Infection | 1 | 3 |
| Lung Cancer | 1 | 0 |
| Sarcoma | 1 | 0 |
| Cardiac illness | 0 | 1 |
| Car accident | 1 | 0 |
| Pneumonia | 0 | 1 |
| Other Cause, Not Specified | 7 | 7 |

**Table S2.** Rates of locoregional and distant recurrence among patients who did and did not discontinue neoadjuvant or adjuvant chemotherapy earlier than planned.

|  | **Early Discontinuation** | |
| --- | --- | --- |
|  | **No**  **n=388**  **No. (%)** | **Yes**  **n=113**  **No. (%)** |
| **Participants with Disease Recurrence** |  |  |
| No recurrence | 359 (92.5) | 104 (92) |
| Yes recurrence | 29 (7.5) | 9 (8.0) |
| Local recurrence only | 8 (2.1) | 3 (2.7) |
| Distant recurrence only | 11 (2.8) | 3 (2.7) |
| Both local and distant recurrence | 7(1.8) | 3(2.7) |
| Unknown location of recurrence | 3(0.8) | 0 (0) |
